# The Chondrogenic Induction Potential for Bone Marrow-Derived Stem Cells between Autologous Platelet-Rich Plasma and Common Chondrogenic Induction Agents: A Preliminary Comparative Study

**DOI:** 10.1155/2015/589124

**Published:** 2015-03-16

**Authors:** Shan-zheng Wang, Qing Chang, Xiang-fei Kong, Chen Wang

**Affiliations:** ^1^Department of Orthopaedics, Zhongda Hospital, Medical School of Southeast University, 87 Ding Jia Qiao Road, Nanjing, Jiangsu 210009, China; ^2^Surgical Research Center, Medical School of Southeast University, 87 Ding Jia Qiao Road, Nanjing, Jiangsu 210009, China

## Abstract

The interests in platelet-rich plasma (PRP) and their application in stem cell therapy have contributed to a better understanding of the basic biology of the prochondrogenesis effect on bone marrow-derived stem cells (BMSCs). We aimed at comparing the effect of autologous PRP with common chondrogenic induction agents (CCIAs) on the chondrogenic differentiation of BMSCs. Rabbit BMSCs were isolated and characterized by flow cytometry and differentiated towards adipocytes and osteoblasts. The chondrogenic response of BMSCs to autologous PRP and CCIAs which included transforming growth factor-*β*1 (TGF-*β*1), dexamethasone (DEX), and vitamin C (Vc) was examined by cell pellet culture. The isolated BMSCs after two passages highly expressed CD29 and CD44 but minimally expressed CD45. The osteogenic and adipogenic differentiation potentials of the isolated BMSCs were also confirmed. Compared with common CCIAs, autologous PRP significantly upregulated the chondrogenic related gene expression, including Col-2, AGC, and Sox-9. Osteogenic related gene expression, including Col-1 and OCN, was not of statistical significance between these two groups. Thus, our data shows that, compared with common chondrogenic induction agents, autologous PRP can be more effective in promoting the chondrogenesis of BMSCs.

## 1. Introduction 

Articular cartilage defects often lead to osteoarthritis and are caused by multiple factors, including genetic, metabolic, biochemical, and biomechanical factors [1]. As a nonvascular tissue, articular cartilage has limited natural ability to repair itself. In recent years, the biological strategies, especially those associated with tissue engineering based on mesenchymal stem cells, hold promising prospects for cartilage repair and regeneration [2]. Bone marrow-derived mesenchymal stem cells (BMSCs) have been widely studied for musculoskeletal tissue repair and regeneration, exhibiting self-renewal and multilineage differentiation potentials [3]. Thus, BMSCs, when induced towards the chondrogenic lineage, hold promising prospect for tissue engineered cartilage. Transforming growth factor-*β*1 (TGF-*β*1), dexamethasone (DEX), and vitamin C (Vc) are commonly applied as the major chondrogenic induction agents (CCIAs) to induce the chondrogenic differentiation of BMSCs.

When constructing the tissue engineered cartilage in vitro, large amounts of qualified seeding cells and CCIAs are needed. However, the exogenous agents are mostly artificial biological agents associated with safety issues when applied in human therapies. Finding an appropriate agent to promote the chondrogenesis of BMSCs is one of the critical factors for cartilage engineering [4].

Platelet-rich plasma (PRP) is a fraction of the autologous whole blood, with the platelet concentration above baseline (platelet concentration of blood) [5]. When activated, the platelets could release a variety of bioactive growth factors, including bone morphogenic protein-2 (BMP-2), connective tissue growth factor (CCN2, also known as CTGF), fibroblast growth factor-2 (FGF-2), growth differentiation factor-5 (GDF-5), and transforming growth factor-*β* (TGF-*β*) [6, 7]. Many of these growth factors present in PRP were reported to promote in vitro proliferation of chondrocytes and chondrogenic differentiation of mesenchymal stem cells [8]. Previous studies have demonstrated the effect of PRP on the chondrogenic differentiation of BMSCs [9–11]. However, till now, comparative results between autologous PRP and CCIAs for the chondrogenic differentiation of rabbit BMSCs are not clear.

To further clarify the significance of PRP in the chondrogenic induction of BMSCs for cartilage engineering, this study investigated the chondrogenic induction potential for BMSCs between autologous PRP and CCIAs.

## 2. Materials and Methods 

All experimental procedures involving animals conformed with the National Institutes of Health Guidelines for the Care and Use of Laboratory Animals and were approved by the Administration Committee of Experimental Animals, Jiangsu Province, China.

### 2.1. Isolation, Culture, and Expansion of BMSCs

BMSCs were isolated from the New Zealand white rabbits as described previously [12]. The isolated BMSCs were seeded in 25 cm^2^ culture flasks (Corning, USA) and cultured in complete basal culture medium, which was composed of low glucose Dulbecco's modified Eagle medium (LG-DMEM) supplemented with 10% fetal bovine serum (FBS), 10 U/mL of penicillin, and 100 mg/mL of streptomycin (all from Gibco) at 37°C under 5% CO_2_ atmosphere. Cells at passage two were used in this study.

### 2.2. Flow Cytometric Analysis

BMSCs at passage two were examined for surface marker expression by flow cytometry. Briefly, the cultured BMSCs were harvested and washed twice with PBS. After centrifugation and removal of the supernatants, the cells were resuspended and incubated with blocking buffer for 30 min at 4°C. Following washing with PBS, the cells were incubated for 30 min at 4°C in the dark with PE-conjugated monoclonal antibodies against CD29, CD44, and CD34 (eBioscience). Flow cytometry analysis was performed with a FACS Calibur cytometer (BD Biosciences) and data were analyzed by CellQuest software.

### 2.3. Evaluation of the Potential for Multilineage Differentiation of BMSCs

The expanded BMSCs were induced toward adipogenic lineage and osteogenic lineage as previously described [13, 14]. As chondrogenic differentiation potential of BMSCs was compared between PRP and CCIAs, the characterization of BMSCs chondrogenesis was not evaluated in this step. Oil Red O staining and Alizarin Red staining were performed to confirm the formation of lipid vesicles and calcium nodule according to previous study [15].

### 2.4. Preparation of PRP

PRP was prepared according to Zhang and Wang [16] with some modifications. Briefly, under general anesthesia, 10 mL fresh blood was obtained from jugular vein of the rabbits using a syringe containing 1.0 mL of acid citrate dextrose-A solution as anticoagulant. The obtained whole blood was primarily centrifuged by a centrifuge (SC-04, Zhongke, China) at 250 g for 10 min. Then, the only plasma fraction was collected and further centrifuged at 1000 g for 10 min. The upper half of the supernatant, accounting for about 40% of the total volume, was removed by suction. The remaining volume (PRP) was stored at −80°C for further use.

### 2.5. Chondrogenesis of BMSCs Induced by PRP and CCIAs

A pellet culture system was used to compare chondrogenesis of BMSCs induced by PRP and CCIAs at 37°C under 5% CO_2_ atmosphere. About 8 × 10^5^ BMSCs were centrifuged at 450 g for 10 min in a 15 mL conical polypropylene tube to form a pellet, which was randomly assigned into PRP group, CCIAs group, and control group, with six pellets for each group. BMSCs pellets in the PRP group were cultured with LG-DMEM containing 10% PRP, 10 U/mL of penicillin, and 100 mg/mL of streptomycin. BMSCs pellets in the CCIAs group were cultured with complete basal culture medium containing TGF-*β*1 (10 *μ*g/L), DEX (10 mmol/L), and Vc (0.05 mmol/L) (all from Sigma). For the control group, BMSCs pellets were only cultured with the complete medium containing 10% FBS. Before the culture, the concentrations of TGF-*β*1 (*n* = 6) from each group were measured by an enzyme-linked immunosorbent assay (ELISA) using a TGF-*β*1 ELISA Kit (R&D Systems). After 3-week culture, pellets were harvested and sectioned for safranin O and alcian blue staining as described previously [17].

### 2.6. Qualitative Real-Time Polymerase Chain Reaction

Total RNA was extracted from cell samples from three groups after a week's culture using Trizol (Invitrogen). Type II collagen (Col-2), aggrecan (AGC), Sox-9, OCN, and type I collagen (Col-1) mRNA levels were measured by qualitative real-time polymerase chain reaction (qRT-PCR) (StepOne Real-Time PCR Applied Biosystems, USA) according to previous studies [18, 19]. Specific primers designed for each cDNA were listed in Table 1.

### 2.7. Statistical Analysis

The statistical significance was determined by one-way analysis of variance (ANOVA). IBM SPSS Statistics 19.0 was used for data analysis. Data was presented as mean ± SD. If there was a significant overall difference between groups, pairwise comparisons were conducted using Scheffe's post hoc test. Values of *P* < 0.05 were considered statistically significant.

## 3. Results 

### 3.1. BMSCs Characterization by Flow Cytometry

**Figure 1:**
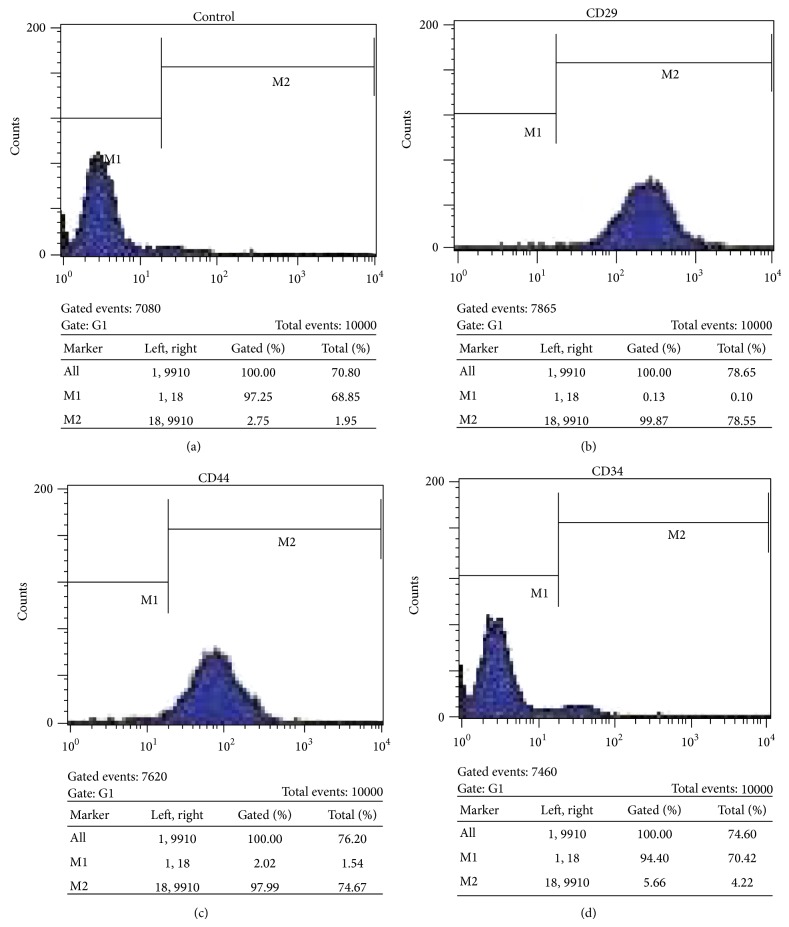
Graphs showing expression of mesenchymal stem cell markers (CD29 and CD44) and hematopoietic stem cell marker (CD34). Filled area shows the expression of target marker. High percentage of cells expressed CD29 and CD44, whereas only few cells expressed CD34.

To characterize the mesenchymal stem cells we isolated from bone marrow, we performed flow cytometric analysis to test the expression of a number of characteristic surface markers. As shown in Figure 1, the isolated cells were positive for CD29 and CD44 and negative for CD34, indicating the typical phenotype of BMSCs.

### 3.2. Osteogenic and Adipogenic Differentiation Potential

**Figure 2:**
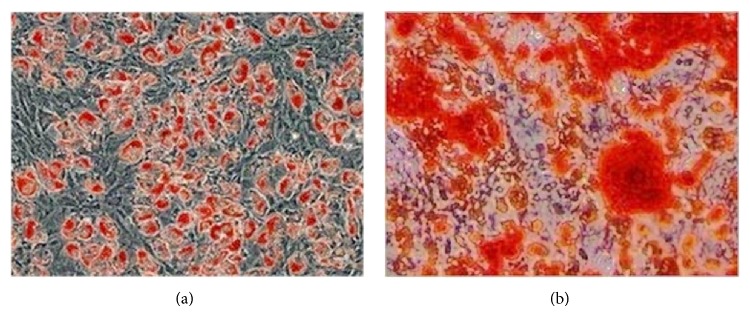
Adipogenic and osteogenic differentiation potential of bone marrow-derived stem cells (BMSCs). Oil Red O staining (a) and Alizarin Red staining (b) were both positive, indicating the adipogenic and osteogenic differentiation potential of BMSCs. Magnification: ×100 (a-b).

After three-week induction, BMSCs were successfully induced toward adipogenic and osteogenic lineages. Lipid droplets were formed and confirmed by Oil Red O staining (Figure 2(a)), indicating the adipogenic differentiation. The osteogenic differentiation potential of the isolated BMSCs was confirmed by Alizarin Red staining, which was positive after osteogenic induction (Figure 2(b)).

### 3.3. Concentrations of TGF-*β*1 in All Groups

**Figure 3:**
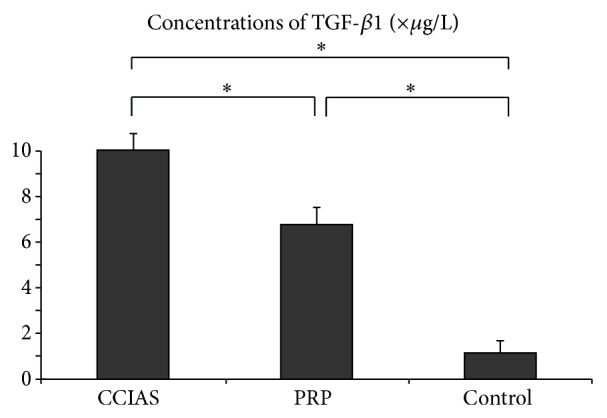
Concentrations of TGF-*β*1 in PRP, CCIAs, and control groups. TGF-*β*1 concentrations in PRP group (*n* = 6), CCIAs group (*n* = 6), and control group (*n* = 6) were measured. The data were expressed as the mean ± error and evaluated by one-way ANOVA. ^*^ *P* < 0.05, with a statistical significance between two groups.

The concentration of TGF-*β*1 was 10.2 ± 1.5 *μ*g/L in CCIAs group, 6.9 ± 1.6 *μ*g/L in PRP group, and 1.2 ± 0.4 *μ*g/L in control group (Figure 3). The concentrations of TGF-*β*1 in PRP and CCIAs groups were significantly higher than that of control group (*P* < 0.05). Among them, CCIAs group had the highest TGF-*β*1 concentration with statistical significance (*P* < 0.05).

### 3.4. Chondrogenesis of BMSCs in PRP and CCIAs Groups

**Figure 4:**
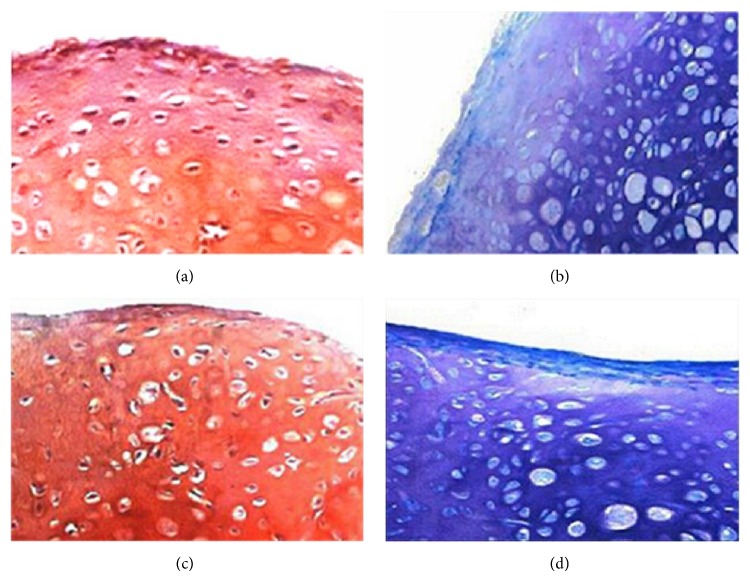
Chondrogenesis of bone marrow-derived stem cells (BMSCs) in PRP and CCIAs groups. Chondrogenic differentiation of BMSCs pellets was confirmed after being cultured in complete medium with PRP or CCIAs for 3 weeks. There was expression of chondrocytes related extracellular matrix as indicated by safranin O (a, c) and alcian blue staining (b, d) for both groups. Magnification: ×200 (a–d).

In PRP and CCIAs groups, the cultured BMSCs pellets became dense and shrank, while the cell pellet of control group collapsed after the culture. After three-week induction, the sections of cell pellets from PRP (Figures 4(a) and 4(b)) and CCIAs (Figures 4(c) and 4(d)) groups were positive for both safranin O staining and alcian blue staining, showing the expression of chondrocytes related extracellular matrix. Both PRP and CCIAs could effectively induce the chondrogenesis of the isolated BMSCs.

### 3.5. Results of Qualitative Real-Time Polymerase Chain Reaction

**Figure 5:**
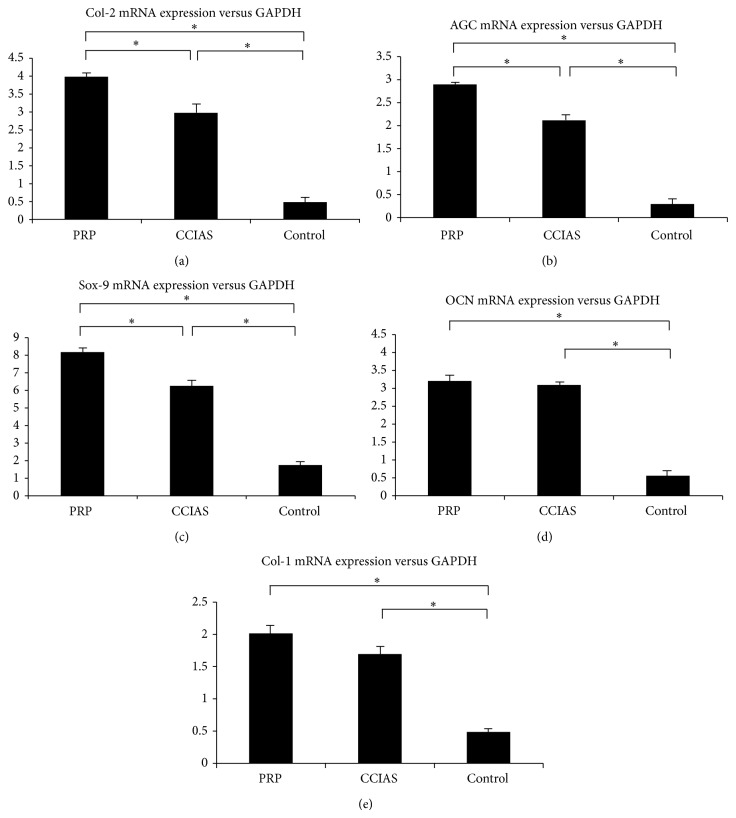
Quantitative analysis of Col-2 (a), AGC (b), Sox-9 (c), OCN (d), and Col-1 (e) gene expression. Statistically significant differences were found by one-way ANOVA in all five genes. ^*^ *P* < 0.05, with a statistical significance between two groups.

The chondrogenesis related mRNA levels of Sox-9, Col-2, and AGC were significantly upregulated in both PRP and CCIAs groups compared to control group (*P* < 0.05) (Figures 5(a)–5(c)). The prochondrogenesis effect of PRP was superior to that of CCIAs with statistical significance (*P* < 0.05). Similarly, the osteogenic related mRNA expression of Col-1 and OCN was significantly higher in PRP and CCIAs groups, compared to control group (*P* < 0.05) (Figures 5(d) and 5(e)). However, Col-1 and OCN expression levels between PRP group and CCIAs group were not of statistical significance (*P* < 0.05).

## 4. Discussion

The use of the growth factors released from the autologous PRP as a powerful substitute for CCIAs has many advantages. The autologous origin of the PRP prohibited disease transmission and immune reaction. Growth factors released from the platelets bound to the receptors of the target cells' membrane instead of entering the cells or their nucleus. Therefore, these growth factors have no mutagenic or tumorigenic effect [20]. The high concentration of platelets from PRP, when activated, could secrete a variety of growth factors, which play a significant role when applied in tissue repair and regeneration [21, 22]. The synergistic effect of multiple growth factors was confirmed to be able to maintain cell proliferation and chondrocyte phenotype of the cultured BMSCs [23]. Therefore, PRP, as the superior autologous active substances, was adopted in this study to compare the prochondrogenesis effect with CCIAs.

A variety of biological factors, such as TGF-*β*1, IGF-1, and FGF, are often applied to induce the chondrogenic differentiation of multiple adult stem cells. Among them, TGF-*β*1 is one of the widely used growth factors for its role in inducing BMSCs toward chondrogenic lineage [23, 24]. In PRP and CCIAs groups, the major prochondrogenesis component was TGF-*β*1, playing a significant role in the chondrogenic differentiation of BMSCs. In this study, the obtained PRP with lower TGF-*β*1 concentration was more effective to induce the chondrogenesis of BMSCs by upregulating the expression of Col-2, Sox-9, and AGC compared to CCIAs, which might be influenced by multiple growth factor “cocktail” effect of PRP. Compared with CCIAs (including TGF-*β*1, DEX, and Vc), PRP is a good alternative. However, PRP and CCIAs are both mixtures, and any component of the mixture may influence the chondrogenesis process of BMSCs.

In this study, we also tested the osteogenic related gene expression, including Col-1 and OCN. Interestingly, we noticed that both PRP and CCIAs upregulated the chondrogenic and osteogenic related gene expression. Col-1 and OCN gene expression was lower in CCIAs group than PRP group, but the statistics were not of significance. Therefore, compared with CCIAs, PRP has a better chondrogenic induction effect without significantly upregulating the osteogenic related mRNA. The “cocktail” effect of growth factors released from PRP also upregulated the osteogenesis related gene expression. However, we noticed the conflictive results that PRP might inhibit osteogenic differentiation of BMSCs [25, 26], which may result from the component and biological activity of various growth factors influenced by different PRP preparation techniques. The interaction of multiple growth factors present in PRP is still unclear and needs further investigations.

In this conclusion, the PRP yielded by our laboratory centrifugation method was potent to induce the chondrogenic differentiation of BMSCs. The chondrogenic induction effect of PRP is more effective than CCIAs without significantly upregulating the expression of osteogenesis related mRNA compared with CCIAs. PRP can be considered in place of CCIAs in cartilage tissue engineering for clinical uses.

## Figures and Tables

**Table 1:** Primer sequences and conditions for qualitative real-time polymerase chain reaction.

Gene	Primer nucleotide sequence	Product size (bp)
Col-2	5′-ATGACAATCTGGCTCCCAACACTGC-3′ (forward)	364
	5′-GACCGGCCCTATGTCCACACCGAAT-3′ (reverse)	

Sox-9	5′-AGCTCACCAGACCCTGAGAA-3′ (forward)	200
	5′-TCCCAGCAATCGTTACCTTC-3′ (reverse)	

AGC	5′-CTTGGGCAGAAGAAAGATCG-3′ (forward)	158
	5′-GTGCTTGTAGGTGTTGGGGT-3′ (reverse)	

OCN	5′-GGTGCAAAGCCCAGCGACTCT-3′ (forward)	199
	5′-GGAAGCCAATGTGGTCCGCTA-3′ (reverse)	

Col-1	5′-cgttgggaccatcatcacc-3′ (forward)	102
	5′-cacccatggacattggaggg-3′ (reverse)	

GAPDH	5′-CTGCCGCCTGGAGAAAG-3′ (forward)	243
	5′-CGACCTGGTCCTCGGTGTA-3′ (reverse)	
